# Differential Dependence on N-Glycosylation of Anthrax Toxin Receptors CMG2 and TEM8

**DOI:** 10.1371/journal.pone.0119864

**Published:** 2015-03-17

**Authors:** Sarah Friebe, Julie Deuquet, F. Gisou van der Goot

**Affiliations:** Faculty of Life Sciences, Global Health Institute, Ecole Polytechnique Fédérale de Lausanne, Lausanne, Switzerland; Van Andel Research Institute, UNITED STATES

## Abstract

ANTXR 1 and 2, also known as TEM8 and CMG2, are two type I membrane proteins, which have been extensively studied for their role as anthrax toxin receptors, but with a still elusive physiological function. Here we have analyzed the importance of N-glycosylation on folding, trafficking and ligand binding of these closely related proteins. We find that TEM8 has a stringent dependence on N-glycosylation. The presence of at least one glycan on each of its two extracellular domains, the vWA and Ig-like domains, is indeed necessary for efficient trafficking to the cell surface. In the absence of any N-linked glycans, TEM8 fails to fold correctly and is recognized by the ER quality control machinery. Expression of N-glycosylation mutants reveals that CMG2 is less vulnerable to sugar loss. The absence of N-linked glycans in one of the extracellular domains indeed has little impact on folding, trafficking or receptor function of the wild type protein expressed in tissue culture cells. N-glycans do, however, seem required in primary fibroblasts from human patients. Here, the presence of N-linked sugars increases the tolerance to mutations in *cmg2* causing the rare genetic disease Hyaline Fibromatosis Syndrome. It thus appears that CMG2 glycosylation provides a buffer towards genetic variation by promoting folding of the protein in the ER lumen.

## Introduction

N-Glycosylation is one of the most prevalent protein modifications and is largely conserved between eukaryotes and prokaryotes [1]. Based on bio-informatics analyses, it has been estimated that more than 50% of all eukaryotic proteins are glycosylated [2] underlining the importance of these modifications in diverse cellular processes including protein folding, stability, trafficking, endocytosis, cell adhesion and cellular recognition events [3].

N-linked protein glycosylation can be viewed as a two-step process with a first step in the endoplasmic reticulum (ER) and the second in the Golgi. In the ER, a core oligosaccharide consisting of Glc_3_Man_9_GlcNac_2_ (Glc: glucose, Man: mannose, GlcNAc: *N-*acetylglucosamine) is attached to asparagine residues within the N-X-S/T consensus sequence and undergoes initial trimming. When the protein is transported to the Golgi, the oligosaccharide undergoes further modifications, in particular the addition of complex sugars [4, 5].

N-glycosylation plays an important role in protein folding in the ER. The core oligosaccharide is attached to the polypeptide chain as it is translocated into the ER lumen across the translocon pore. The two outer glucoses are immediately removed by glucosidases I and II. This enables association of the nascent polypeptide chain with calnexin or calreticulin, chaperones involved in ER folding and quality control [6, 7]. These two ER-resident lectins specifically bind monoglucosylated oligomannose glycans [8]. If folding and, when relevant, oligomerization are successful, the newly synthesized protein exits the ER via COPII coated vesicles and is subsequently routed to its final destination [9].

If folding is aberrantly delayed or unsuccessful, the protein can be targeted to ER-associated degradation (ERAD) [10]. Prolonged presence in the ER leads to extensive mannose trimming of the oligosaccharide, which acts as a targeting signal for ERAD [11–13]. Exposed mannoses are recognized by the ERAD lectins OS-9 and XTP3-B [14–16]. Since degradation is proteasome mediated, ERAD substrates must be retrotranslocated to the cytoplasm through a ubiquitination-mediated process [17]. Once in the cytosol, the remaining glycans are removed by a glycanase, before the protein is handed over to the proteasome for degradation [18, 19]. Thus N-linked sugars play crucial roles first in promoting protein folding via glucose residues and then targeting the protein to degradation via the mannose residues.

We here investigated the importance of N-glycosylation in the trafficking and function of two surface receptors probably involved in the homeostasis of the extracellular matrix, namely TEM8 (tumor endothelial marker 8) and CMG2 (capillary morphogenesis gene 2). TEM8 and CMG2 are two highly homologous type I membrane proteins, composed of an extracellular von Willebrandt A domain (60% identity between the two proteins), an Ig-like domain, a transmembrane domain and a cytosolic tail that differs in size between the two proteins but is initiated by a 68% identical domain (S1 Fig.).

TEM8 was discovered as an upregulated gene in human tumor endothelium [20]. It was subsequently found to serve as a receptor for anthrax toxin, hence the name anthrax toxin receptor 1 (ANTXR1) [21]. Recent studies describe TEM8 as a cancer [22–24] and cancer stem cell [25] marker due to its upregulation in tumor, but not physiological, vasculature. Consistently, interfering with TEM8 leads to a decrease in tumor angiogenesis [26–28]. Mutations in TEM8 can lead to GAPO syndrome, a rare, complex and severe autosomal-recessive disorder [29, 30].

CMG2 was found as the second most upregulated gene in a 3D culture of endothelial cells [31]. Its vWA domain was found to bind *in vitro* to the ECM proteins laminin and collagen type IV [31]. CMG2 knockout mice display an accumulation of ECM in different organs [32, 33], indicating a role for CMG2 in the homeostasis of ECM. This notion is strengthened by the pathology of patients afflicted with Hyaline Fibromatosis Syndrome, a disease caused by mutations in *cmg2*. Patients suffer from the accumulation of a hyaline material in skin and other organs which can be life threatening or highly debilitating [34]. Most reported mutations in the vWA and the Ig-like domain of CMG2 lead to misfolding of the protein, provoking its retention in the ER and its degradation by ERAD, resulting in loss of protein function [35]. The drastic effect of these point mutations suggests a sensitive folding landscape for CMG2.

Both TEM8 and CMG2 contain potential N-glycosylation sites: N166 and N184 in the vWA and N262 in the Ig-like domain of TEM8, and N250 and N260 in the Ig-like domain of CMG2. In this study, we show that glycosylation can occur at all potential sites and that glycosylation is necessary for TEM8 to fold, exit the ER and reach the plasma membrane. We found that CMG2 is less dependent on glycosylation for folding and thus its N-glycosylation-independent exit from the ER depends on the folding capacity/ER quality control stringency of the cell. Importantly, the dependence on glycosylation becomes apparent when genetic mutations such as those found in Hyaline Fibromatosis Syndrome patients, decrease folding efficiency of the extracellular domains.

## Materials and Methods

### Cells and reagents

HeLa cells were grown in Modified Eagle’s medium (MEM) (Invitrogen, Carlsbad, CA) supplemented with 10% fetal calf serum, 2 mM L-glutamine, non-essential amino acids, penicillin and streptomycin. RpeI cells, patient fibroblasts and Flp-In T-REx 293 CMG2 cells (Invitrogen, Carlsbad, CA) were grown in Dulbecco’s modified Eagle’s medium (DMEM) supplemented with 10% fetal calf serum and 2 mM penicillin and streptomycin. For induction of CMG2, Flp-In T-REx 293 CMG2 cells were treated for 24h with 0.1 μg/ml doxycycline.

The human CMG2 (isoform 4, Uniprot P58335–4) gene, with a V5 epitope at the C-terminus was cloned in the pcDNA3.1/V5-HIS-TOPO expression vector (Invitrogen, Carlsbad, CA) and was provided by J. Martignetti (Mount Sinai School of Medicine, New York, NY; Dowling et al., 2003). For stable cell lines, CMG2 was cloned into the pcDNA5/FRT/TO vector (Invitrogen, Carlsbad, CA) and transfected cells were selected according to the manufacturer’s protocol. The human TEM8 (isoform 1, Uniprot Q9H6X2–1) gene with an HA epitope was cloned in the pIREShyg2 vector (Liu and Leppla, 2003). Mutations were generated using the QuikChange II XL Site-Directed Mutagenesis Kit (Agilent, Santa Clara, CA). All plasmids were transfected into cells using Fugene according to the manufacturer’s protocol (Promega, Madison, WI).

Anthrax toxin was purified as described before [36]. Polyclonal goat antibody (#771B) against Protective Antigen (PA) was from List Biological Laboratories (Campbell, CA) and used at 1/2000 dilution, monoclonal mouse V5 antibody (#R960–25) was from Invitrogen (Carlsbad, CA) and used at 1/2000 dilution; monoclonal rat HA-HRP antibody (#12 013 819 001) was from Roche (Basel, Switzerland) and used at 1/2000 dilution; monoclonal mouse Ubiquitin antibody (#sc-8017) from Santa Cruz Biotechnology Inc. (Santa Cruz, CA) and used at 1/500 dilution; polyclonal rabbit calnexin antibody was produced by Eurogentec for our lab [37] and used at 1/2000 dilution; polyclonal rabbit BiP antibody (#ab21685) from Abcam (Cambridge, UK) used at 1/1000 dilution; polyclonal rabbit antibody against TEM8 was generated in our lab and used at 1/2000 and polyclonal goat CMG2 antibody (#AF2940) was from R&D Systems (Minneapolis, MN) and used at 1/2000 dilution.

HRP-conjugated secondary antibodies were from Pierce Chemical Co. (Rockford, IL) and Alexa-conjugated secondary antibodies from Molecular Probes (Invitrogen, Carlsbad, CA). Streptavidin-agarose conjugated beads were from Sigma-Aldrich (St. Louis, MO), protein G beads were from GE Healthcare (Uppsala, Sweden) and HA-beads from Roche (Basel, Switzerland);

Treatments with N-glycosidase and Endoglycosidase H (New England Biolabs, Ipswich, MA) were performed as previously described [38].

### Immunofluorescence

Transiently transfected HeLa cells were fixed with 4% paraformaldehyde and permeabilized with 0.1% Triton X100, and stained with antibodies against the V5 or HA tag and the ER marker BiP followed by an appropriate secondary antibody. Images were acquired using a Plan-Apochromat 63x/1.4 oil objective on a Zeiss LSM 710 (Carl Zeiss Microimaging, Thornwood, NY), equipped with an Axiocam MRm camera using the Zen 2009 acquisition software. FIJI and Adobe Illustrator software were used to prepare the figures.

### Surface biotinylation and immunoprecipitation

For immunoprecipitation, confluent cells were washed three times with PBS. Cells were lysed by incubation for 30 min at 4°C with 0.5% NP-40, 500 mM Tris-HCl, pH 7.4, 20 mM EDTA, 10 mM NaF, 30 mM sodium pyrophosphate decahydrate, 2 mM benzamidine, 1 mM PMSF, 1 mM NEM and a cocktail of protease inhibitors (Roche, Basel, Switzerland). Cells lysates were incubated overnight at 4°C with anti-V5 antibody and protein G sepharose beads for CMG2, or HA-beads for TEM8. For the ubiquitination assay, cells were treated for 4h with 10 μM MG132 or 0.1 μM Bafilomycin A1 (Sigma) before cell lysis.

For surface biotinylation, confluent cells were incubated with 0.2 mg/ml NHS-SS-biotin (Pierce) in PBS for 30 min at 4°C and washed 3x 10 min with sterile PBS containing 100 mM NH_4_Cl. Cells were lysed and the lysate was immunoprecipitated with streptavidin-coated agarose beads. After SDS-PAGE and Western blotting against the V5 or HA antibody, biotinylated CMG2 or TEM8 proteins and expression level were quantified with Image Quant TL 2005 / Typhoon software (GE Healthcare) or BioID software (Fusion). Biotinylated proteins values were normalized to expression level values. Expression of mutants at the cell surface was normalized to that of the WT protein. To assess surface expression of non-glycosylated endogenous protein, cells were treated with 5 μg/ml tunicamycin (Sigma) for 16h and then labeled as described above.

For *in vivo* anthrax protective antigen (PA) binding, transiently transfected HeLa cells were incubated for 1h at 4°C with 500 ng/ml PA^83^ in internalization medium (IM medium), (Glasgow minimal essential medium, Invitrogen, 10mM HEPES, pH 7.4). Cells were washed twice with warm IM medium to remove excess toxin and incubated for 10 min at 37°C to induce cleavage and heptamerization. Cells were then lysed and immunoprecipitated against TEM8-HA or CMG2-V5. For *in vitro* PA binding, transiently transfected HeLa cells were lysed and the lysate was incubated for 1h at 4°C with 1μg/ml PA^83^. Lysates were immunoprecipitated against TEM8-HA or CMG2-V5.

### Graphics

Molecular graphics and analyses were performed with the UCSF Chimera package [39]. For TEM8, the graphic is based on structure 3N2N from the Protein Data Bank (http://www.rcsb.org/pdb/). For CMG2, the graphic is based on structure 1TZN from PDB and on modeling from [40].

### Ethical Statement

Primary Human fibroblasts from a control and a Hyaline Fibromatosis syndrome patient were obtained with patient consent, research using these cells was approved by the “Commission Cantonale d’éthique de la recherche sur l’être humain” and are registered under the approval No A070055 of the Swiss Federal Office of the Environment. Patients provided written consent that patient derived cells could be used in any studied aimed at a better understanding of Hyaline Fibromatosis syndrome and the gene involved. The patient signed a standard consent form approved by the ethics committee.

## Results

### TEM8 and CMG2 can undergo N-glycosylation on all predicted sites

**Fig 1:**
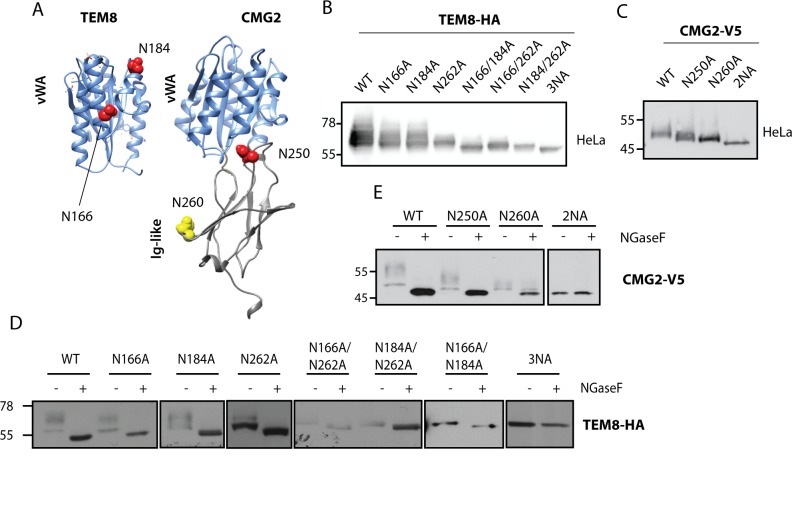
CMG2 and TEM8 can undergo N-glycosylation on all predicted sites. **A)** Graphics depicting glycosylation sites on TEM8 and CMG2. Sites in red are unique to the respective proteins, N260 in CMG2 (yellow) corresponds to N262 in TEM8. **B)** Expression of TEM8 glycosylation mutants in HeLa cells. Cells were transfected for 48h with the respective cDNAs. Expression was analyzed by SDS-PAGE and Western Blotting. **C)** Expression of all CMG2 glycosylation mutants in HeLa cells. Cells were transfected for 48h with the respective cDNAs. Expression was analyzed by SDS-PAGE and Western Blotting. **D)** Endoglycosidase F (NGaseF) treatment on TEM8 glycosylation mutants. 40 μg of cell extracts were treated or not with NGaseF and analyzed by SDS-PAGE and Western Blotting. **E)** Endoglycosidase F (NGaseF) treatment on CMG2 glycosylation mutants. 40 μg of cell extracts were treated or not with NGaseF and analyzed by SDS-PAGE and Western Blotting.

TEM8 and CMG2 have been shown to be glycosylated, yet the location and number of sites have not been determined [38]. They respectively contain 3 and 2 predicted N-glycosylation sites, of which only one is conserved: N262 in TEM8 corresponding to N260 in CMG2 within the Ig-like domain (Fig. 1A). To test whether these sites can be modified, we generated single, double and triple asparagine mutants. Expression of the mutants was analyzed by transient transfection of HeLa cells (Fig. 1BC). For both TEM8 and CMG2, mutation of a single asparagine residue, at whatever position, was sufficient to induce a change in electrophoretic mobility. The most dramatic change was observed when mutating the conserved site in the Ig-like domain (N262/N260, Fig. 1BC). Mobility increased as more asparagines were mutated. Also, TEM8 and CMG2, which migrate as smears when WT, migrated as a well-defined single band as glycosylation sites were mutated (Fig. 1BC). All together this analysis indicates that all sites can be modified in WT TEM8 and CMG2.

To confirm that all predicted sites can indeed be glycosylated, Total Cell Extracts (TCE) of cells expressing the mutants were treated with N-Glycosidase F (NGaseF), an enzyme that removes all glycan side chains of a protein, regardless of their modification or localization [41]. For both TEM8 and CMG2, NGaseF-treatment led to a change in electrophoretic mobility of WT and all mutants, with the exception of the mutants with all potential sites mutated (Fig. 1DE), indicating that all sites can be modified *in vivo*. Note that all sites might not be modified simultaneously, and thus cells might express differentially glycosylated species.

### The number of glycan sidechains and their localization determine trafficking efficiency of the protein

**Fig 2:**
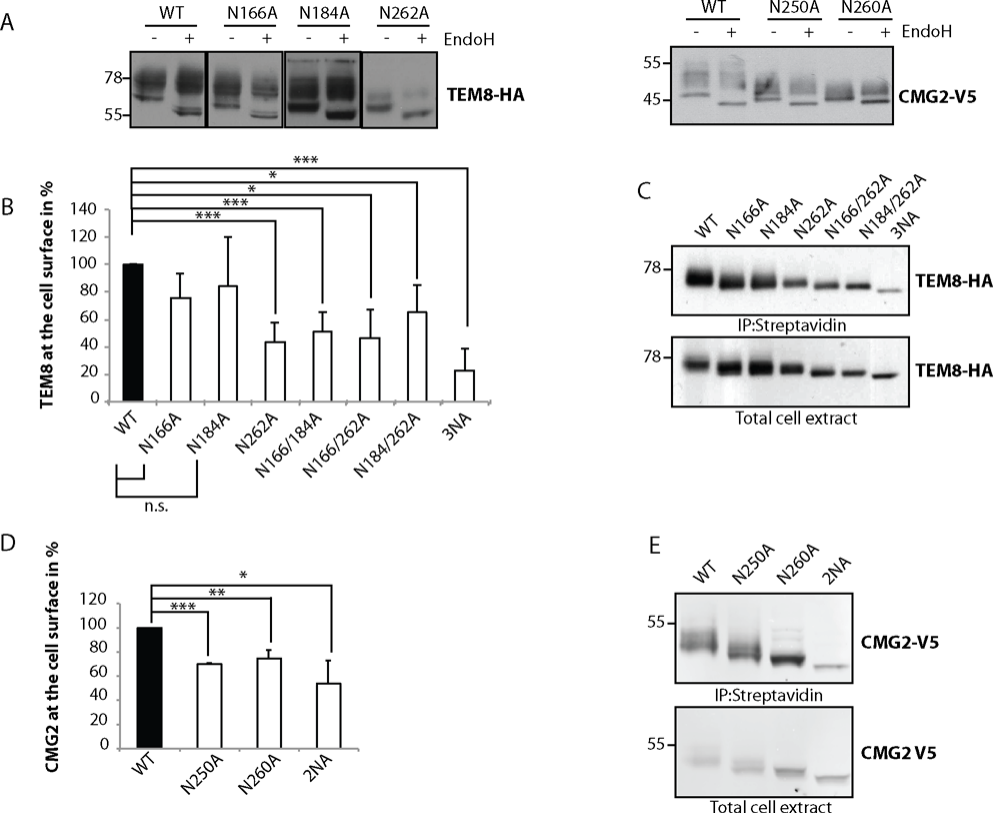
Number and localization of glycan sidechains determine trafficking efficiency of TEM8 and CMG2. **A)** Endoglycosidase H (EndoH) treatment on TEM8 and CMG2 single mutants. HeLa cells were transfected for 48h with the respective cDNAs. 40 μg of cell extracts were treated or not with EndoH as described before. Samples were analyzed by SDS-PAGE and Western Blotting. **B)** Quantification of surface biotinylation experiments to determine amount of TEM8 at the cell surface. All mutants were corrected for their expression levels and then normalized to WT, which was set at 100%. Errors represent standard deviation. Statistics were calculated using an unpaired t-test. n ≥ 3. * p≤0.05, ** p≤0.01, *** p≤0.001 **C)** Representative Western Blots of surface biotinylation. HeLa cells were transfected 48h with the respective cDNAs. Proteins at the cell surface were labeled with biotin, immunoprecipitated with streptavidin beads and blotted against TEM8-HA. **D)** Quantification of surface biotinylation experiments to determine amount of CMG2 at the cell surface. All mutants were corrected for their expression levels and then normalized to WT, which was set at 100%. Errors represent standard deviation. Statistics were calculated using an unpaired t-test. n ≥ 3. * p≤0.05, ** p≤0.01, *** p≤0.001 **E)** Representative Western Blots of surface biotinylation. HeLa cells were transfected 48h with the respective cDNAs. Proteins at the cell surface were labeled with biotin, immunoprecipitated with streptavidin beads and blotted against CMG2-V5.

The first hurdle for any protein is to fold. Because glycosylation has been shown to promote folding of certain proteins such as the multi-pass membrane protein cystic fibrosis transmembrane conductance regulator CFTR [42] or human immunodeficiency virus-1 (HIV-1) protein gp120 [43], we investigated whether mutating glycosylation sites in CMG2 and TEM8 affects their folding. As a readout, we utilized the modification of N-linked sugars by Golgi enzymes since proper folding is a prerequisite for ER exit and trafficking to the Golgi apparatus. Modification of N-linked sugars by Golgi enzymes renders them insensitive to the enzyme Endoglycosidase H (EndoH). Transiently expressed WT TEM8 or CMG2 migrate as a smear, corresponding to the Golgi modified form, and a lower molecular weight band, corresponding to the immature ER form, which is EndoH sensitive [35, 38]. All single CMG2 and TEM8 glycosylation mutants migrated as an EndoH insensitive smear and an EndoH sensitive band, indicating that a fraction of all mutants is able to fold correctly, exit the ER and reach the Golgi (Fig. 2A). Fully glycosylation deficient mutants cannot be analyzed using this assay.

Following sugar modification in the Golgi, CMG2 and TEM8 are transported to the plasma membrane where they exert anthrax toxin receptor and presumably physiological functions. To assess whether glycosylation influences surface targeting, we performed a surface biotinylation assay. This assay allows the affinity isolation of the surface population and comparison to the overall expression levels. Mutation of either of the two vWA domain glycosylation sites in TEM8, N166 and N184, did not affect surface expression when compared to WT, but simultaneous mutation of both led to a 50% decrease in surface expression (Fig. 2BC). A similar drop in surface expression was observed when mutating N262 in the Ig-like domain (Fig. 2BC). A pronounced drop in surface expression was observed when all three sites where modified (Fig. 2BC). Thus TEM8 requires two N-linked glycans for efficient trafficking to the cell surface, of which one site must be N262.

For CMG2, loss of either of the two glycosylation sites within the Ig-like domain lead to a 30% drop in plasma membrane targeting (Fig. 2DE). Surface targeting was also impaired when both sites were absent (Fig. 2DE), yet at least 50% was still properly addressed to the plasma membrane.

Thus plasma membrane targeting of both TEM8 and CMG2 is enhanced by the presence of two N-linked sugars, one of which must reside in the Ig-like domain.

### TEM8 glycosylation promotes ER exit

**Fig 3:**
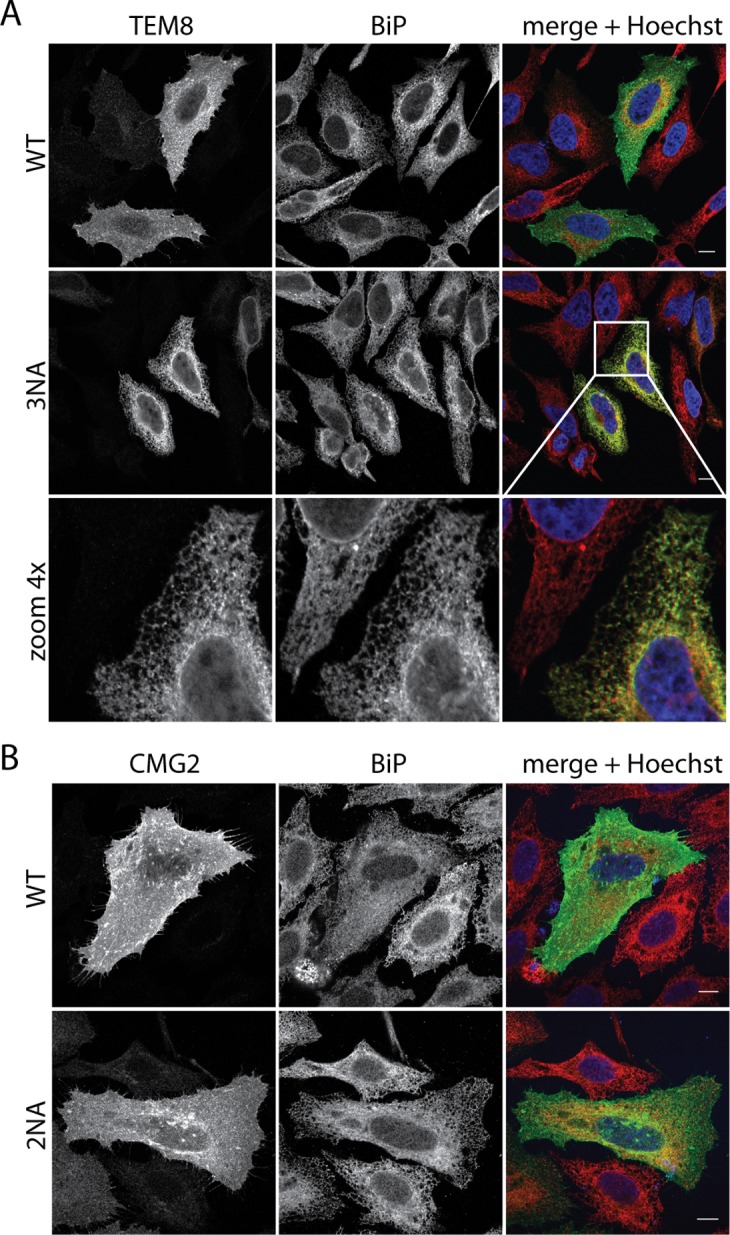
Localization of TEM8 and CMG2 glycosylation mutants. **A)** Immunofluorescence of transiently transfected HeLa cells. Cells were transfected for 48h with the respective cDNAs. Cells were fixed, permeabilized and stained for TEM8-HA, endogenous BiP and Hoechst. Scalebars represent 10 μm. **B)** Immunofluorescence of transiently transfected HeLa cells. Cells were transfected for 48h with the respective cDNAs. Cells were fixed, permeabilized and stained for CMG2-V5, endogenous BiP and Hoechst. Scalebars represent 10 μm.

The surface biotinylation analysis indicates that fully glycosylation-deficient TEM8 and CMG2 do not or only partially reach the plasma membrane. We analyzed their localization by immunofluorescence microscopy. As expected, the WT form of TEM8 and CMG2 could be detected at the plasma membrane as well as intracellular structures (Fig. 3AB and S2 Fig.). Plasma membrane staining was also observed for CMG2 2NA (Fig. 3B and S2 Fig.). Note that immunofluorescence does not allow a quantitative analysis of surface expression as does surface biotinylation (Fig. 2). In contrast, the triple 3NA TEM8 mutant was absent from the plasma membrane and showed clear ER staining, co-localizing with the ER chaperone BiP (Fig. 3A).

### Non-glycosylated TEM8 is an ER quality control and ERAD substrate

**Fig 4:**
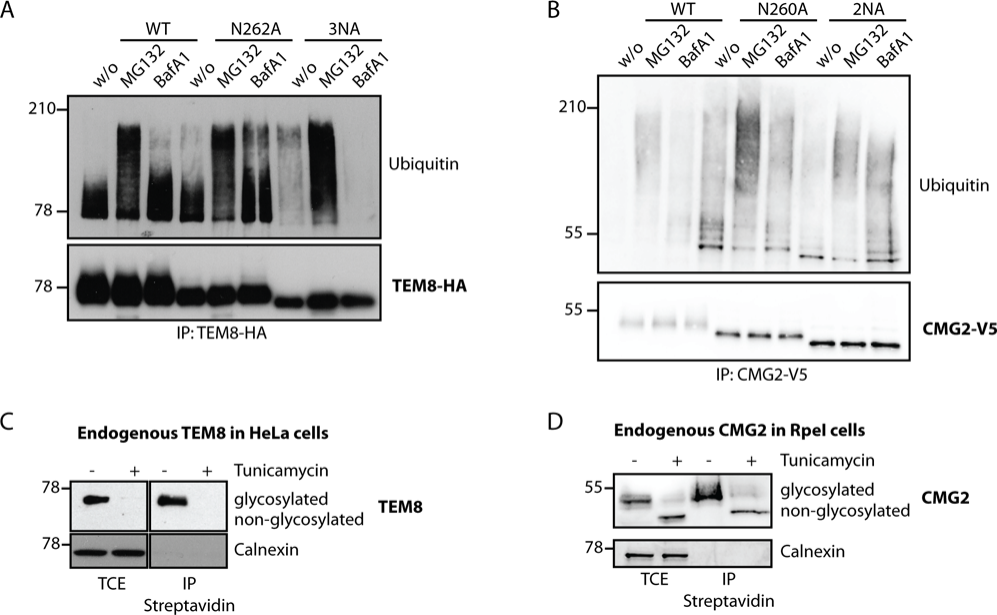
Non-glycosylated TEM8 is an ER quality control and ERAD substrate. **A)** HeLa cells were transfected for 48h with the respective cDNAs. Cells were treated or not with MG132, an inhibitor of the proteasome or Bafilomycin A1, a drug preventing endosomal acidification and thus lysosomal degradation. Immunoprecipitates against TEM8-HA were analyzed by SDS-PAGE and Western Blotting against Ubiquitin and TEM8-HA. **B)** HEK cells stably expressing CMG2 under the control of a tetracycline inducible promotor were induced for 24h with 0.1μg/ml doxycycline. Cells were treated or not with MG132 or Bafilomycin A1. Immunoprecipitates against CMG2-V5 were analyzed by SDS-PAGE and Western Blotting against Ubiquitin and CMG2-V5. **C)** HeLa cells were treated or not with tunicamycin, an antibiotic blocking the co-translational transfer of glycan sidechains in the ER by blocking the oligosaccharyltransferase (OST) for 16h. Surface proteins were labeled with biotin and immunoprecipitates against streptavidin were analysed for TEM8 or Calnexin as a negative control. **D)** RpeI cells were treated or not with tunicamycin for 16h. Surface proteins were labeled with biotin and immunoprecipitates against streptavidin were analysed for CMG2 or Calnexin as a negative control.

The above observations indicate that glycosylation promotes ER exit, raising the possibility that in the full absence of glycosylation, folding in the ER is impaired, leading to recognition of the protein by the ER quality control and possible targeting to ERAD. To address this issue, we monitored the ubiquitination status of TEM8 and CMG2, since this post-translation modification is required for targeting the protein to the proteasome. Since ubiquitination might lead to rapid degradation, rendering the species undetectable, we treated cells with the proteasome inhibitor MG132. We also monitored the effect of Bafilomycin A1, which inhibits acidification of the endosome, thereby preventing lysosomal protein degradation, since this is the second major protein degradation route in the cell. Inhibitors were used only for a few hours to minimize the potential secondary effects on protein translation for example. For both WT and the N262A mutant of TEM8, a limited smear of ubiquitination was observed, which was enhanced by Bafilomycin A1 treatment, consistent with an ubiquitination-mediated targeting of TEM8 from the plasma membrane to lysosomes [38]. MG132 treatment led, as expected, to the appearance of a long smear, a hallmark of polyubiquitination, the targeting signal for the proteasome (Fig. 4A). These observations are consistent with the fact that upon transient expression of WT TEM8, the majority is targeted to the cell surface, from where degradation occurs in lysosomes, while a smaller a fraction remains in the ER and is degraded by the proteasome (Figs. 1 and 2). When a similar analysis was performed for the 3NA TEM8 mutant, no effect was observed upon Bafilomycin A1 treatment, consistent with the absence of TEM8 3NA at the plasma membrane. However a long smear, revealing polyubiquitination, was observed under control conditions and strongly enhanced upon MG132 treatment (Fig. 4A, lane 8). All together this analysis indicates that the 3NA TEM8 mutant is retained in the ER and targeted to the ERAD pathway. Note that while the majority of WT TEM8 folds and exists the ER, targeting to ERAD is also observed indicating that the efficiency of TEM8 folding is not 100%.

For CMG2, the ubiquitination pattern of WT and mutants was qualitatively similar, in all cases sensitive to both drugs, with a higher molecular weight smear upon MG132 treatment and a lower molecular weight smear upon neutralization of lysosomes (Fig. 4B). This behavior is consistent with our finding that a significant percentage (50%, Fig. 2D) of even the 2NA mutant reaches the plasma membrane.

The above experiments indicate that non-glycosylated TEM8 is retained in the ER and potentially an ERAD substrate, while CMG2, under these experimental conditions, is less dependent on glycosylation for its proper expression.

The above experiments were performed in HeLa or HEK cells transiently expressing TEM8 or CMG2 WT and glycosylation mutants and allowed us to reveal a differential sensitivity to glycosylation. Different cell types however have different capacities of protein folding and ER quality control [44]. Also overexpression might saturate the ER capacity to promote folding of the overexpressed protein of interest. We therefore next monitored endogenously expressed proteins, TEM8 in HeLa cells (which do not express CMG2) and CMG2 in Rpe1 cells [45, 46]. N-glycosylation was prevented by treating cells for 16h with tunicamycin, a drug inhibiting the transfer of the core oligosaccharide to the nascent polypeptide chain by oligosaccharyltransferase (OST). Tunicamycin treatment led to a complete loss of TEM8 protein (Fig. 4C). Even after surface biotinylation and enrichment of surface proteins with streptavidin beads, TEM8 was undetectable. In contrast, expression of CMG2 was not significantly modified by tunicamycin (Fig. 4D). The CMG2 band migrated at a lower molecular weight as expected from the absence of glycosylation. Importantly, non-glycosylated CMG2 was transported to the cell surface as revealed by surface biotinylation. Since tunicamycin disrupts N-glycosylation of all proteins in the cell, the loss of TEM8 could potentially be due to gross secondary effects. This is however unlikely given the fact that these results are fully consistent with the surface biotinylation experiments performed on the glycosylation mutants and the lack of effect of tunicamycin on CMG2, which is highly homologous to TEM8. These experiments confirm that endogenous TEM8 is highly dependent on glycosylation for its expression, while in Rpe1 cells, endogenous CMG2 does not significantly rely on glycosylation for its plasma membrane targeting.

### Loss of glycosylation affects binding of Anthrax toxin to TEM8 but not to CMG2

Since glycosylation is not required for targeting of CMG2 to the plasma membrane, we next investigated whether it is required for its ability to bind its ligand. Parallel experiments were performed for TEM8. We used protective antigen (PA), the receptor binding subunit of the anthrax toxin, as a surrogate ligand. As a negative control, we used the D50A mutants of both receptors, which harbors a mutation in the ligand-binding domain and therefore cannot bind PA [47, 48].

**Fig 5:**
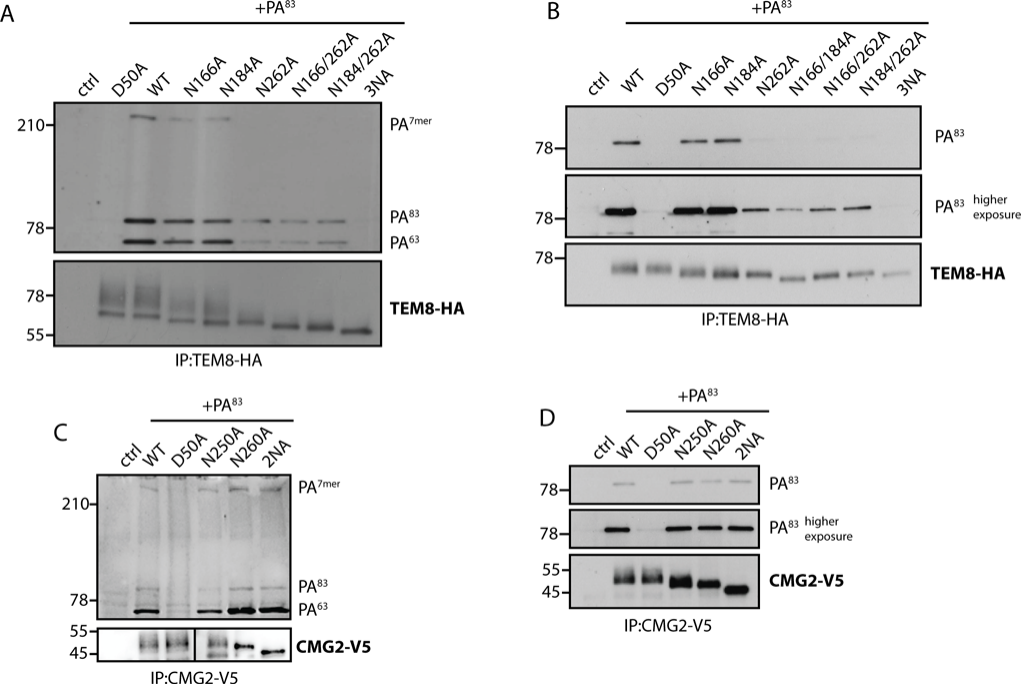
Loss of glycosylation affects binding of Anthrax toxin to TEM8 but not to CMG2. **A and C)** HeLa cells were transfected for 48h with the respective cDNAs. Cells were treated for 1h at 4°C with 500 ng/ml PA^83^ and shifted to 37°C for 10 min to induce cleavage and heptamerization. Immunoprecipitates against TEM8-HA/CMG2-V5 were analyzed by SDS-PAGE and Western Blotting against PA and TEM8-HA/CMG2-V5. Control cells are non-transfected. D50A is a binding deficient mutant that serves as a negative control. **B and D)** HeLa cells were transfected for 48h with the respective cDNAs. Cells were lysed and incubated for 1h at 4°C with 1 μg/ml PA^83^. Immunoprecipitates against TEM8-HA/CMG2-V5 were analyzed by SDS-PAGE and Western Blotting against PA and TEM8-HA/CMG2-V5. Control cells are non-transfected. D50A is a binding deficient mutant that serves as a negative control.

We monitored binding of full-length PA (PA^83^) to the receptor, the processing to its 63 kDa form (PA^63^) and the conversion of the 63 kDa form into an SDS-resistant oligomeric form (PA^7mer^), using SDS-PAGE and Western Blotting. The presence of oligomers indicates that PA has been transported to endosomes, where the conversion to the membrane inserted SDS-resistant form occurs [49]. Upon addition of PA to cells expressing any of the single TEM8 mutants, we observed a decrease in PA binding, cleavage, and heptamer formation, as compared to WT. Only monomers were observed in immunoprecipitates of the N262A mutant (Fig. 5A). This could be due either due lower binding, impaired oligomerization or enhanced release of the oligomer from the receptors [50]. The triple, fully glycosylation-deficient TEM8 mutant failed to bind PA (Fig. 5A), not surprising given its ER localization. Since differences observed in this assay might, at least partly, be due to a decrease in surface expression of the mutants, we next monitored PA binding *in vitro*. In this assay, PA is added to cell lysates, bypassing the localization issue [35]. Of the single TEM8 mutants, only N262A showed a severe decrease in binding when compared to the WT. This was also true for double mutants and PA binding became essentially undetectable for the triple asparagine TEM8 mutant (Fig. 5B). Thus, glycosylation of TEM8, in particular at position 262, is required for efficient PA binding. Recombinant, bacterial expressed, TEM8 vWA domain is fully competent for PA binding [51], thus glycosylation *per se* is not required for ligand binding. Therefore the absence of PA binding to the 3NA mutant is likely due to a defect in folding in the cellular context in the absence of glycosylation, which would be consistent with the full ER retention of TEM8 3NA. In contrast, for CMG2, both *in vivo* and *in vitro* binding assays showed no significant difference in binding, cleavage, or heptamer formation between WT and mutant proteins partially or fully deficient in glycosylation (Fig. 5CD).

### Glycosylation acts as a buffer for CMG2 ectodomain mutations

**Fig 6:**
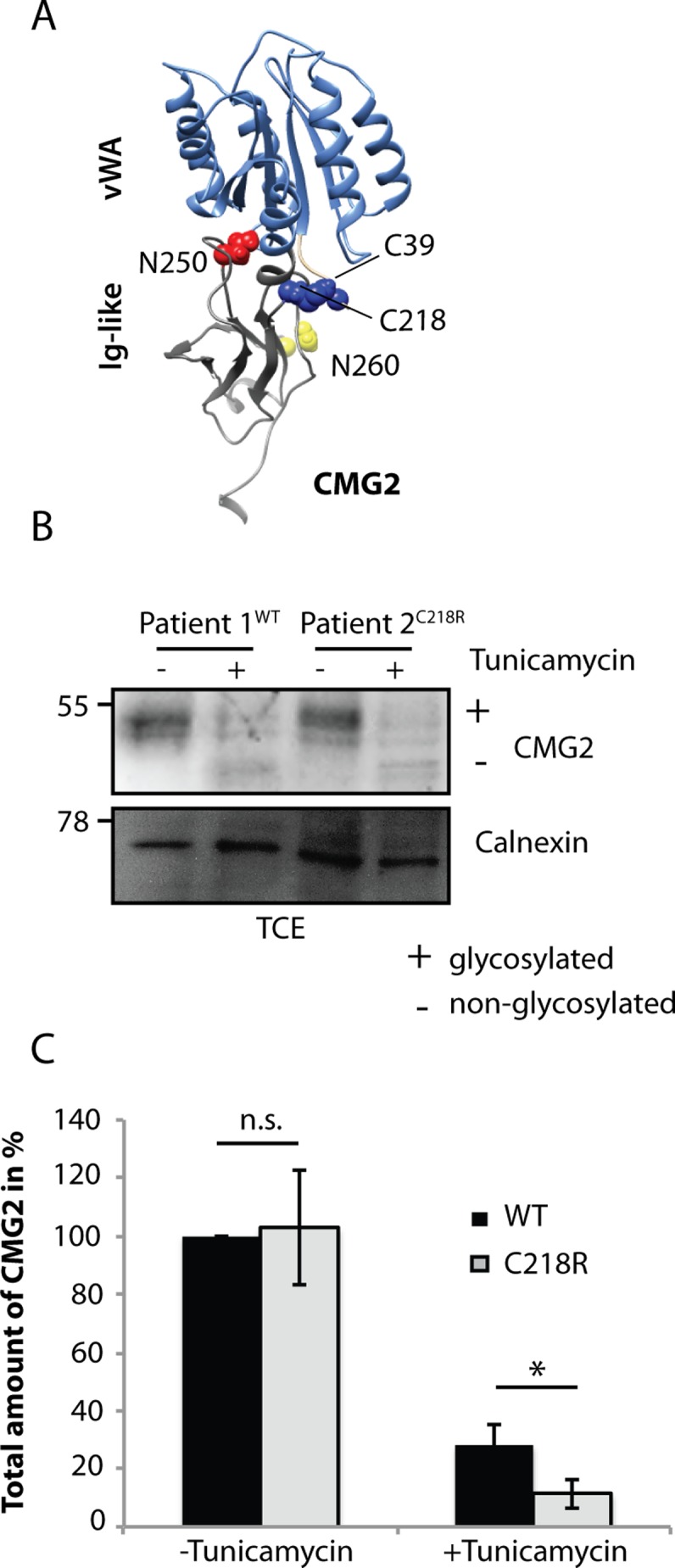
Glycosylation acts as a buffer for CMG2 ectodomain mutations. **A)** Graphic showing the disulfide bridge C39-C218 (blue) present in CMG2 WT **B)** Fibroblast cells were treated or not with tunicamycin for 16h and TCE were analyzed by SDS-PAGE and Western Blot. Representative Western Blot with control fibroblasts and patient fibroblasts. Calnexin serves as a loading control. **C)** Quantification of total protein levels. CMG2 levels are normalized to WT protein level without tunicamycin treatment, which was set to 100%. Statistics were calculated using an unpaired t-test. Errors represent standard deviation. n ≥ 3. * p≤0.05

Our study reveals that in Rpe1 cells or upon transfection into HeLa cells, CMG2 is only mildly sensitive to the loss of N-glycosylation. Our analyses of Hyaline Fibromatosis Syndrome (HFS) mutations have however revealed that CMG2 is rather sensitive to ectodomain mutations in terms of folding [35, 40]. Indeed most ectodomain missense mutations were found to lead to defects in ER folding and targeting to ERAD [35, 40]. We therefore hypothesized that N-glycosylation of CMG2 might favor folding in the presence of protein destabilizing mutations and thus serve as a “buffer” for genetic variation. To test this hypothesis, we searched for a HFS inducing mutation that localizes to the ectodomain of CMG2 but does not affect plasma membrane targeting. This is a rare situation since we have found that most ectodomain mutations lead to CMG2 degradation by ERAD [35, 40]. We found a homozygous patient carrying the c.652T>C mutation which leads to p.C218R. Modification of Cys-218 leads to the disruption of the disulfide bond that links the N and C-termini of the vWA domain (Fig. 6A). We have previously shown that upon transient overexpression in tissue culture cells, the C218R mutant has a partial folding defect, leading to a 60% drop in surface expression yet its ability to bind PA is not impaired [35, 40]. Instead of transient expression in tissue culture cells, we could analyze patient cells. As shown in Fig. 6, CMG2 is expressed at the same level in cells from the C218R expressing patient as in cells from a control patient. Also the migration pattern of C218R CMG2 was similar to that of WT CMG2, indicating that sugar modifications and in particular addition of complex sugars in the Golgi had occurred. To investigate the importance of CMG2 glycosylation in patient-derived fibroblasts, cells were treated with tunicamycin. In marked contrast to what we observed in the tissue culture cell line Rpe1, expression of WT CMG2 dropped by 70% upon tunicamycin treatment indicating that in these primary human fibroblasts, glycosylation was required for efficient expression of WT CMG2. Consistently, tunicamycin also led to a drastic drop of the C218R CMG2 HFS variant (Fig. 6BC). Interestingly, the comparison of WT to C218R CMG2 expression under conditions of tunicamycin treatment showed that C218R expression was almost three fold lower than that of WT. Thus C218R is in fact a protein destabilizing mutation, but this is no longer apparent in the presence of glycosylation, a situation in which its expression is similar to that of WT. Thus consistent with our hypothesis, glycosylation appears to overcome the folding defect generated by the C218R mutation. As opposed to most other ectodomain mutations, C218R does not lead to a major loss of protein expression at the cell surface but to a loss of protein function, which we are currently addressing.

## Discussion

TEM8 and CMG2 are two transmembrane surface glycoproteins involved in homeostasis of the extracellular matrix but can also act as anthrax toxin receptors. Mutations in the *tem8* or *cmg2* genes may lead to two severe genetic diseases, GAPO syndrome [29, 30] and Hyaline Fibromatosis syndrome, respectively [34]. While the genetic origin of GAPO syndrome has only recently been identified and thus no genotype-phenotype studies have yet been reported, our studies on the HFS mutations indicate that the disease is due to a loss of protein function, often as a consequence of the loss of protein expression [40]. Depending on the specific patient mutations, the defects of CMG2 expression were attributed to premature mRNA degradation or impaired folding in the ER. This led us to investigate the potential role of N-glycosylation on the folding and subsequent trafficking of TEM8 and CMG2.

We show that both proteins can undergo N-glycosylation on all of their predicted sites, three in TEM8 and two in CMG2, of which only one is conserved and localizes to the Ig-like domain. Transient overexpression experiments in tissue culture cells reveal that optimal expression is achieved when all sites are available to be modified. The requirement for glycosylation was however less stringent for CMG2 since a significant fraction of the protein was found to exit the ER, indicating proper folding, reach the plasma membrane and bind its ligand in the absence of N-linked sugars. In contrast, fully glycosylation-deficient TEM8 was retained in the ER and failed to bind anthrax PA toxin, together suggesting that folding was impaired. TEM8 was in fact affected by the loss of any of its glycosylation sites, and losses were to a large extent additive.

That sugars promote protein folding, trafficking and function has been reported for a variety of proteins, as reviewed in [3, 52]. Proteins that depend on glycosylation for folding and other functions are diverse and range from mammalian to viral and bacterial proteins [53, 54]. Depending on where the glycosylation sites are localized in proteins, sugars can have a local or a global impact on the protein, i.e. by influencing the secondary structure in the vicinity of the modified residue [55, 56]. The presence of sugars affects the hydrophilicity of the protein but also provides the opportunity to interact with lectin chaperones such as calnexin, further increasing the positive effect on folding. The present work leads to the unexpected finding that two proteins that are very closely related and show a high degree of sequence identity/similarity (S1 Fig.), differ significantly in their dependence towards glycosylation.

The tolerance of CMG2 toward the lack of glycosylation was however only apparent in certain cell types (HeLa and Rpe1 tested here). In primary human fibroblasts however, glycosylation of CMG2 appeared essential for proper expression, since tunicamycin led to a 70% drop in expression. This differential behavior likely reflects the difference in expression of the folding and quality control arsenal expressed by these cells. It has indeed been reported that the expression of chaperone and folding enzymes greatly differs between cells and tissues [44]. It could thus be that primary cells have a poorer folding capacity, a more stringent ER quality control and/or a more potent ERAD pathway, and that under these conditions optimal folding of CMG2 can only be achieved in the presence of N-linked glycans.

Moreover, glycosylation appears to provide a buffer towards genetic variation. Glycosylation would render CMG2 more tolerant to mutations that fail to fold without the assistance of lectin chaperones. Consistent with this hypothesis, we found that in the absence of glycosylation, C218R CMG2 was expressed at 30% of WT, whereas under normal glycosylation conditions, expression was similar to WT.

That chaperones can act as buffers for genetic variations has previously been shown. This might not only help proteins that have acquired a mutation to fold, but could also be a driver for genetic variation and evolution [57–59]. Our results now suggest a variation on this theme: by being modified with glycan side chains, a protein is more prone to interact longer or more often with lectin chaperones. This in turn would help the protein to fold correctly even if it harbors a destabilizing mutation. The glycosylated form of the protein would be more tolerant to mutations while not affecting or even promoting function. One could even envision that some mutants have a beneficial increased occupancy of the glycosylation sites. It was indeed observed that excessive N-glycosylation of Aquaporin 2 mutants increased their stability and promoted folding, thereby partly preventing their degradation [60]. The importance of N-glycosylation in protecting TEM8 and CMG2 will be further tested, as mutations in the encoding genes identified in GAPO and HSF patients will be reported.

## Supporting Information

S1 Fig Alignment of human TEM8 and CMG2. Sequence alignment using Jalview [61] for human TEM8 (ANTR1) isoform 1 and CMG2 (ANTR2) isoform 4. Indicated are the domains of the proteins as well as the glycosylation sites (asterisks).
(TIF)

S2 Fig Immunofluorescence of TEM8 WT and CMG2 WT and N260A. Images shown in Fig. 3 of TEM8 WT, CMG2 WT and N260A with an additional zoomed image.
(TIF)

S3 Fig Original blots of Figs. 1, 2 and 4. Uncropped blots shown in Figs. 1,2 and 4. Indicated is the area that was used for the figures.
(TIF)

S4 Fig Original blots of Figs. 5 and 6. Uncropped blots shown in Figs. 5 and 6. Indicated is the area that was used for the figures.
(TIF)
