# Supplementary figures and images for: Differential Dependence on N-Glycosylation of Anthrax Toxin Receptors CMG2 and TEM8
Source: PLoS One. 2015 Mar 17;10(3):e0119864. doi: 10.1371/journal.pone.0119864 (PMC4363784; doi:10.1371/journal.pone.0119864)

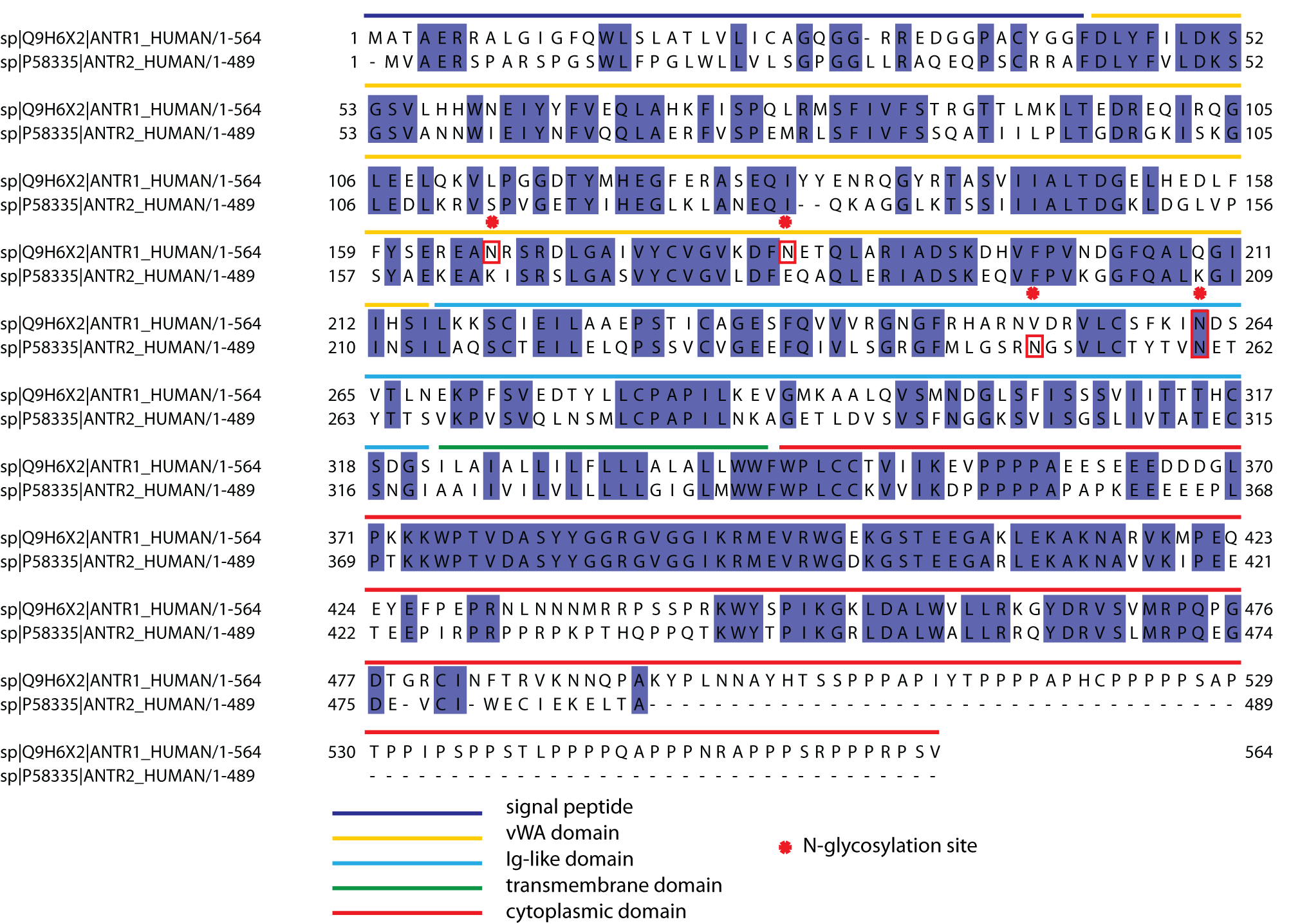

Supplement: S1 Fig — Sequence alignment using Jalview [61] for human TEM8 (ANTR1) isoform 1 and CMG2 (ANTR2) isoform 4. Indicated are the domains of the proteins as well as the glycosylation sites (asterisks). (TIF) [file pone.0119864.s001.tif]

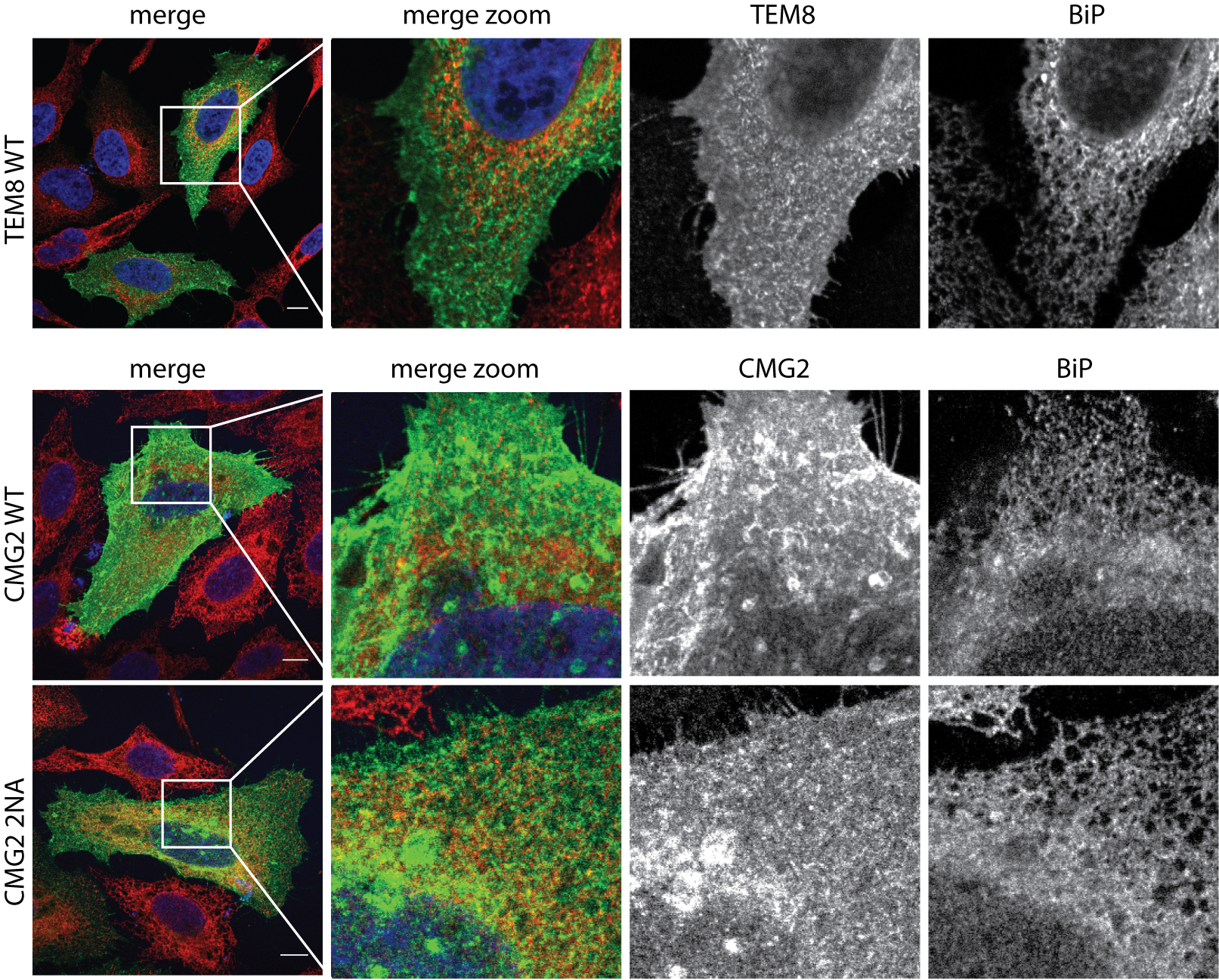

Supplement: S2 Fig — Images shown in Fig. 3 of TEM8 WT, CMG2 WT and N260A with an additional zoomed image. (TIF) [file pone.0119864.s002.tif]

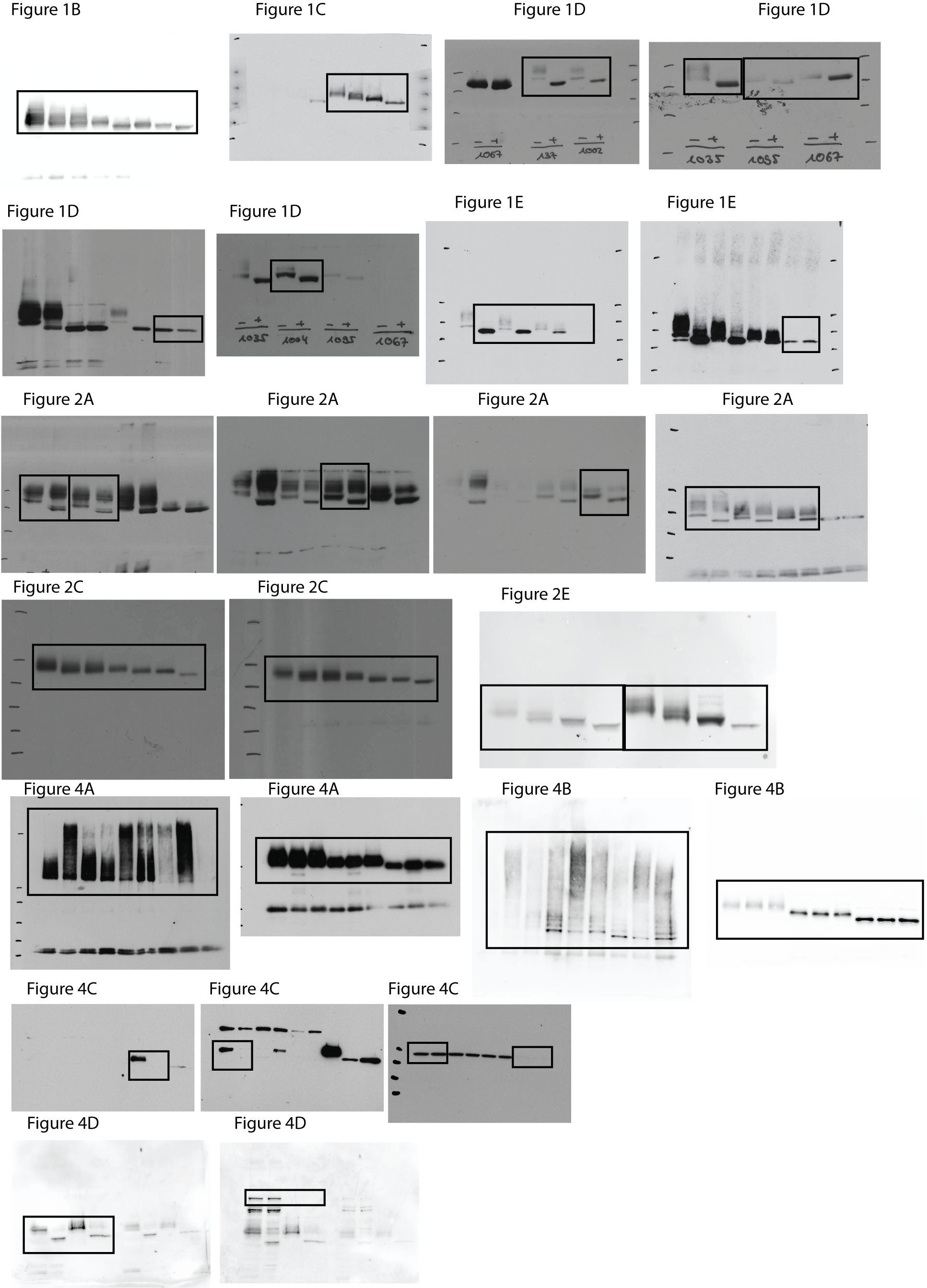

Supplement: S3 Fig — Uncropped blots shown in Figs. 1,2 and 4. Indicated is the area that was used for the figures. (TIF) [file pone.0119864.s003.tif]

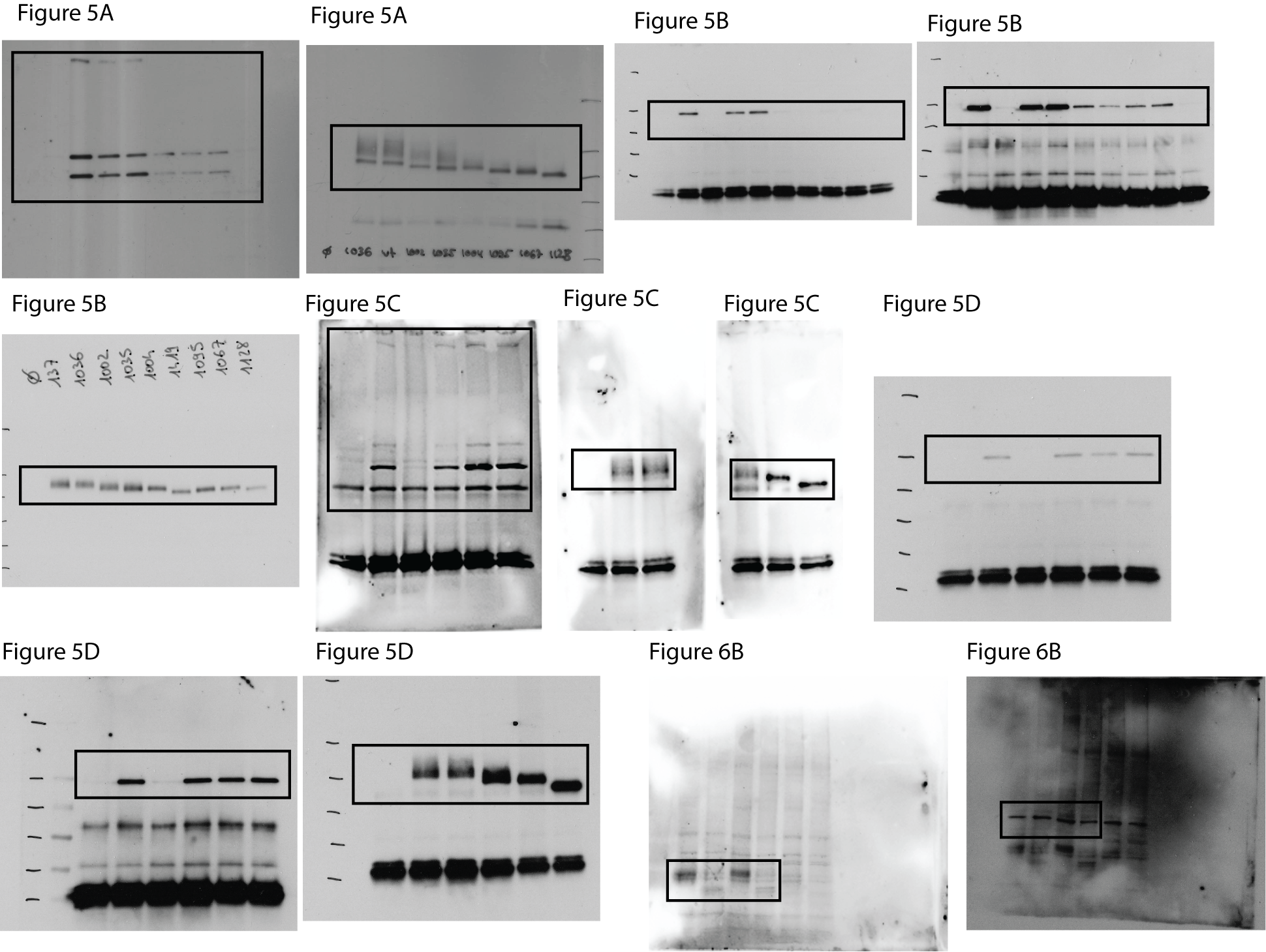

Supplement: S4 Fig — Uncropped blots shown in Figs. 5 and 6. Indicated is the area that was used for the figures. (TIF) [file pone.0119864.s004.tif]
